# Genome-Wide Investigation and Analysis of Microsatellites and Compound Microsatellites in *Leptolyngbya*-like Species, Cyanobacteria

**DOI:** 10.3390/life11111258

**Published:** 2021-11-18

**Authors:** Dan Yao, Lei Cheng, Lianming Du, Meijin Li, Maurycy Daroch, Jie Tang

**Affiliations:** 1 Key Laboratory of Coarse Cereal Processing, Ministry of Agriculture and Rural Affairs, Chengdu University, Chengdu 610106, China; yaodancdu1113@163.com (D.Y.); dulianming@cdu.edu.cn (L.D.); 2 Beijing Engineering and Technology Research Center of Food Additives, Beijing Technology & Business University, Beijing 100048, China; chenglei@btbu.edu.cn; 3 School of Environment and Energy, Peking University Shenzhen Graduate School, Shenzhen 518055, China; 1801213472@pku.edu.cn (M.L.); m.daroch@pkusz.edu.cn (M.D.)

**Keywords:** *Leptolyngbya*, microsatellites, compound microsatellites, motif, cyanobacteria

## Abstract

Microsatellites (simple sequence repeats, SSRs) are ubiquitously distributed in almost all known genomes. Here, the first investigation was designed to examine the SSRs and compound microsatellites (CSSRs) in genomes of *Leptolyngbya*-like strains. The results disclosed diversified patterns of distribution, abundance, density, and diversity of SSRs and CSSRs in genomes, indicating that they may be subject to rapid evolutionary change. The numbers of SSRs and CSSRs were extremely unevenly distributed among genomes, ranging from 11,086 to 24,000 and from 580 to 1865, respectively. Dinucleotide SSRs were the most abundant category in 31 genomes, while the other 15 genomes followed the pattern: mono- > di- > trinucleotide SSRs. The patterns related to SSRs and CSSRs showed differences among phylogenetic groups. Both SSRs and CSSRs were overwhelmingly distributed in coding regions. The numbers of SSRs and CSSRs were significantly positively correlated with genome size (*p* < 0.01) and negatively correlated with GC content (*p* < 0.05). Moreover, the motif (A/C)_n_ and (AG)_n_ was predominant in mononucleotide and dinucleotide SSRs, and unique motifs of CSSRs were identified in 39 genomes. This study provides the first insight into SSRs and CSSRs in genomes of *Leptolyngbya*-like strains and will be useful to understanding their distribution, predicting their function, and tracking their evolution. Additionally, the identified SSRs may provide an evolutionary advantage of fast adaptation to environmental changes and may play an important role in the cosmopolitan distribution of *Leptolyngbya* strains to globally diverse niches.

## 1. Introduction

*Leptolyngbya* are ecologically important cyanobacteria that are often found to be prosperous in thermal environments [1,2]. *Leptolyngbya* strains have shown extensive biotechnological potential in pharmaceutical [3] and biodegradation applications [4]. Although an increasing number of *Leptolyngbya* strains were proposed, the identification of *Leptolyngbya*-like strains has been controversial due to their simple morphology. The heterogeneity of *Leptolyngbya* has been questionable since the establishment of this genus [5]. Moreover, the genus *Leptolyngbya* is recognized as polyphyletic [6,7], and a taxonomic reevaluation is essential for better understanding this genus. To compensate for the limited information provided by cell morphology, molecular approaches have been widely applied to establish the correct taxonomy. Molecular markers, primarily 16S rRNA and/or 16S-23S intergenic spacer (ITS), alleviate the taxonomic recognition to some extent. However, it was ineffective in dealing with closely related *Leptolyngbya* species [8]. As the increasing availability of genome sequences, bioinformatic analyses at the genomic level may provide new insight into the evolutionary relationship among *Leptolyngbya* species.

Microsatellites, also called simple sequence repeats (SSRs), are tandem repeats with a length of 1–6 bp in genomes [9]. SSRs are characterized as hypervariability and hypermutability [10]. They appeared to be scattered across the genome and be traced in both coding and non-coding regions [11]. SSRs are crucial in the determination and understanding of microbial genome evolution [12]. Moreover, SSRs are important evolutionary markers that are useful in tracking SSRs length variations such as point mutations, duplications, DNA repair, and replication slippage across the entire genomes [12]. In addition, an increasing number of examples illustrate that bacteria can exploit this instability of SSRs as potential engines of genetic variability and bacterial adaptation on short evolutionary time scales without an increased overall mutation rate [13]. Moreover, SSRs are of significant interest to researchers in light of substantial applications in genetic mapping, DNA fingerprinting, population genetics, gene regulation, paternity studies, and evolution [9,14,15].

Compound microsatellites (CSSRs) are more complex sequences typically consisting of two or more SSRs, e.g., (GCA)_n_-(C)_n_-(CA)_n_, and are supposed to possess higher polymorphism than SSRs [16]. Jakupciak and Wells [17] suggested that compound microsatellites resulted from recombination between homologous SSRs. Diverse genomic features and evolutionary traces of CSSRs were reported between related species, e.g., *Escherichia coli* and lactobacilli [18].

With the development of sequencing technology and in silico methodologies, conventional SSR mining based on genomic libraries is being replaced by computational mining from tremendous genome sequences [19,20]. The increasing availability of genome sequences and specialized bioinformatics software tremendously accelerate the characterization of SSRs on a genome-wide scale, which obviously is a prerequisite for understanding their distribution, predicting their function, and tracking their evolution.

To date, there were numerous *Leptolyngbya* genomes available according to the genomic resources of the National Center for Biotechnology Information (NCBI), offering an opportunity for SSR discovery at the genomic level. To our knowledge, a genome-wide survey of SSRs and CSSRs is unavailable for *Leptolyngbya* genomes. The present study was designed to mine and analyze SSRs and CSSRs, and to further reveal the patterns of distribution, abundance, density, and diversity of SSRs and CSSRs in *Leptolyngbya* genomes. This study provides the first insight into SSRs and CSSRs in *Leptolyngbya* genomes and may be useful for future studies on the function and evolution of these repeat sequences in *Leptolyngbya* strains.

## 2. Materials and Methods

### 2.1. Genome Sequences

According to the genomic resources of NCBI at the time of this study (20 October 2020), a total of 53 *Leptolyngbya* genomes were retrieved as a dataset for SSR and CSSR analysis. Information regarding these genomes was summarized in Table S1. Filtering was further performed to remove obvious non-*Leptolyngbya* strains by literature search and Blast search of 16S rRNA sequence against NCBI database. In addition, genomic annotations of these *Leptolyngbya* genomes were also downloaded for corresponding analysis.

To illustrate the relationship among the strains studied, multi-locus sequence analysis (MLSA) was performed using concatenated sequences of 15 genes from each genome. These genes were *frr*, *pgk*, *rplA*, *rplB*, *rplC*, *rplE*, *rplK*, *rplL*, *rplM*, *rplN*, *rplP*, *rplT*, *rpmA*, *rpsC*, and *rpsS*. Genes were recommended locus for MLSA by reference [21] and selected based on a larger dataset with more genes given to the availability and completeness in genomes. Strains with much less common genes to other genomes were filtered for phylogenetic analysis. Sequences of each gene were aligned, edited, and trimmed in Mega7 [22]. Sequences were concatenated using BioEdit 7 [23]. Maximum-Likelihood (ML) phylogenetic analyses were carried out using PhyML v3.0 [24]. Parameter settings in PhyML were followed as described [25]. The whole-genome average nucleotide identity (ANI) between genomes was calculated using the ANI calculator with default settings [26].

### 2.2. Identification and Analysis of SSRs and CSSRs

The perfect SSR and CSSRs were identified in each genome using the repeat search engine Krait v1.2.2 [27]. In light of small genomes in *Leptolyngbya* strains, the minimum repeats were customized to 6, 3, 3, 3, 3, and 3 for mono-, di-, tri-, tetra-, penta-, and hexanucleotide SSRs, respectively [16]. The maximum distance allowed between any two adjacent SSRs (*d*max) was set to 10 bp for the CSSRs analysis. The other parameters in Krait were maintained as default. All identified perfect SSRs and CSSRs were mapped into coding and non-coding regions to feature coordinates using Krait. The complexity and motifs of CSSRs were investigated as well.

To mitigate the effect of genome size on the comparative analysis, the numbers of SSRs and CSSRs were normalized as relative abundance (RA), the number of SSRs and CSSRs per kb of the genome sequence studied, and relative density (RD), the total length contributed by each SSRs and CSSRs per kb of the genome sequence studied.

### 2.3. Statistical Analysis

To facilitate interpretation, statistical terms used in this study were abbreviated as follows. nSSR: number of SSRs in each genome; nCSSR: number of CSSRs in each genome; cSSR: individual SSR being part of such a CSSR; C: complexity defined by the number of cSSRs in a CSSR; ncSSR: number of cSSR in each genome; and cSSR%: percentage of ncSSR account for nSSR in each genome (cSSR% = ncSSR/nSSR).

The Pearson correlation coefficient (ρ) was calculated using a custom R script to uncover the associations between variables, including genome size, GC content, nSSR, nCSSR and ncSSR. Significance levels of 0.05 and 0.01 were applied. The significance of CSSR representation in each genome was statistically evaluated by an index, Z [28]. Z scores were computed using the following equations:

(1) $$\bar{C}=\frac{1}{n}\overset{n}{\underset{i=1}{\sum }}\left(\frac{{\mathrm{ncSSR}}_{i}}{{\mathrm{nCSSR}}_{i}}\right)$$

(2) $${\mathrm{nCSSR}}_{\exp}=\frac{{\mathrm{ncSSR}}_{i}}{\bar{C}}$$

(3) $$Z=\frac{\left({\mathrm{nCSSR}}_{\mathrm{obs}}-{\mathrm{nCSSR}}_{\exp}\right)}{\sqrt{{\mathrm{nCSSR}}_{\exp}}}$$

where n, number of genomes studied (n = 46); i, genome order; ${\mathrm{ncSSR}}_{i}$, number of cSSR in genome; nCSSR_i_ (also called nCSSR_obs_), observed number of CSSRs in genome; $\bar{C}$, average of complexity of 46 genomes ($\bar{C}$ = 2.069 in this study); nCSSR_exp_, expected number of CSSRs in genome.

## 3. Results

### 3.1. Phylogenetic Relationship of Leptolyngbya Strains

**Figure 1:**
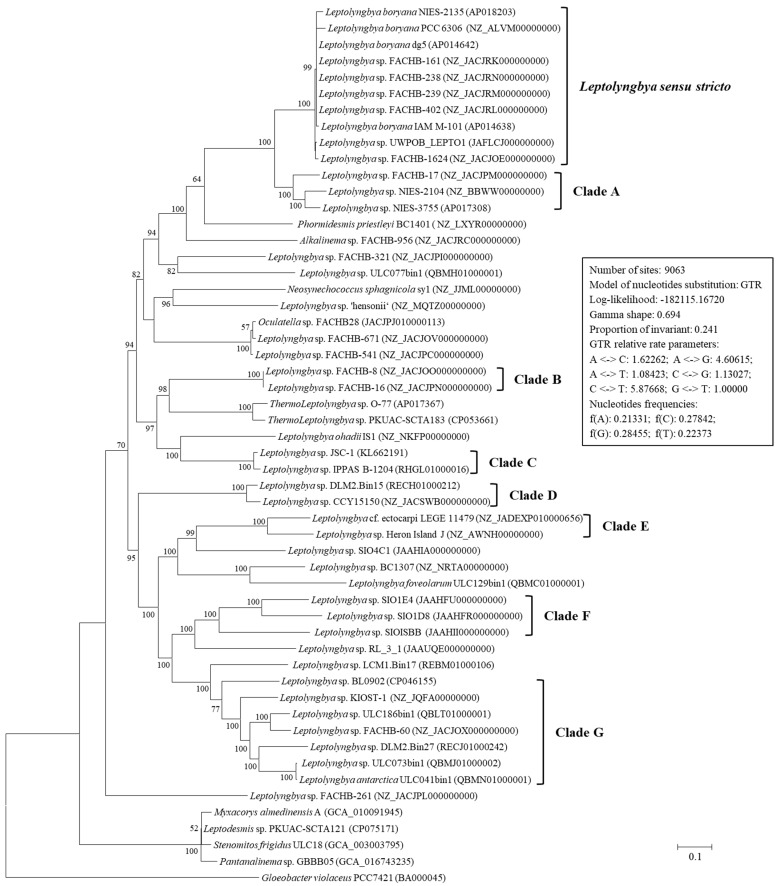
Maximum-likelihood phylogenomic tree of concatenated protein alignment of 15 genes shared by all genomes. Bootstrap values are indicated at nodes. Scale bar = 10% substitutions per site.

Four strains were filtered from analysis for new genus proposal [29], generating a dataset comprising 49 genomes of *Leptolyngbya*-like strains. Based on the availability and quality of a single locus from each genome, the concatenated sequences of 15 genes representing 43 *Leptolyngbya*-like strains and 11 reference strains were constructed to infer the phylogenetic relationship. The reference strains contained strains from the family Leptolyngbyaceae and Oculatellaceae, including *Alkalinema*, *Leptodesmis*, *Myxacorys*, *Neosynechococcus*, *Oculatella*, *Pantanalinema*, *Phormidesmis*, *Stenomitos* and *Thermoleptolyngbya*, and *Gloeobacter* as outgroup. The ML phylogenetic tree (Figure 1) inferred by the concatenated sequences categorized the cyanobacterial strains into well-supported clades. Ten strains were grouped into previously established genus *Leptolyngbya sensu stricto* [30], and the other *Leptolyngbya*-like strains were scattered across the tree (Figure 1). Noticeably, *Leptolyngbya* sp. FACHB-671 and FACHB-541 are closely clustered with *Oculatella* sp. FACHB28, while *Leptolyngbya* sp. FACHB-261 was an outlier (Figure 1). This result indicated evident conflict between phylogeny and taxonomy, thus removing the three genomes from subsequent analysis. The ML tree (Figure 1) and distance matrix of the concatenated sequences (Table S2) revealed that considerably genetic divergences existed between strains within *Leptolyngbya sensu stricto* and the other *Leptolyngbya*-like strains. The *Leptolyngbya* strains did not form a monophyletic clade but mixed with the reference strains (Figure 1), suggesting the interspecific heterogeneity within this genus. This result is not unexpected since it is well-known that *Leptolyngbya* is polyphyletic [6]. However, further ANI analysis showed that the ANI values between *Leptolyngbya sensu stricto* and the other *Leptolyngbya*-like strains were all below 80% (Table S3), which conformed to the typical values (<80% ANI) for organisms of different genera [31]. Taken together, it is still quite challenging to determine the actual taxonomy of these *Leptolyngbya*-like strains without more evidence, e.g., morphology, etc. Thus, dedicated works in separate studies are required to illustrate the taxonomy positions of these *Leptolyngbya*-like strains with polyphasic approaches. For the convenience of presentation and comparisons, the *Leptolyngbya*-like strains were grouped according to the phylogenetic tree (Figure 1) and the ANI values (Table S3) for genus delimitation.

### 3.2. Number, Relative Abundance and Density of SSRs and CSSRs

A total of 46 genomes of *Leptolyngbya*-like strains were finalized for SSR and CSSR analysis. These strains were originated from diverse habitats (Table 1), including freshwater, marine, hot spring, terrestrial, and other environments. Across the 46 genomes of *Leptolyngbya*-like strains, a total of 741,115 perfect SSRs were identified (Table 1). Extremely uneven distribution of SSRs number was observed among genomes, ranging from 11,086 to 24,000. The relative abundance (RA) and relative density (RD) of SSRs both showed significant dissimilarity among the genomes of *Leptolyngbya*-like strains, shifting from 2.00 to 3.32/kb and from 13.20 to 24.08 bp/kb. The RA and RD of genus *Leptolyngbya sensu stricto* varied from 2.47 to 2.53/kb and from 16.30 to 16.67 bp/kb, respectively. The RA and RD values of genus *Leptolyngbya sensu stricto* were higher than that of Clade A, C, and D but lower than that of Clade B, E, F, and G. The RA and RD values of other ungrouped *Leptolyngbya*-like strains were also higher or lower than that of genus *Leptolyngbya sensu stricto*.

There were 24,703 CSSRs identified in the 46 genomes of *Leptolyngbya*-like strains (Table 1). Similar to SSRs, the number of CSSRs tremendously varied among genomes, from 286 to 907. Massive variations were also exhibited by RA and RD of CSSRs (Table 1), ranging from 0.05 to 0.14/kb and from 0.61 to 2.11 bp/kb, respectively. Analogously, strains within genus *Leptolyngbya sensu stricto* or clades showed accordant RA and RD of CSSRs. Genus *Leptolyngbya sensu stricto*, together with clade C and D, showed similar RA and RD of CSSRs, the values of which were higher than that of Clade A and lower than that of Clade B, E, F, and G (Table 1).

The number of cSSR in each genome (ncSSR) ranged from 580 to 1865 (Table 1). And the results suggested that only a small part of all SSRs (less than 10%) in each genome consisted of a compound motif as revealed by cSSR% (Table 1). Between genus *Leptolyngbya sensu stricto* and clades, different cSSR% was observed, e.g., 5.88–6.42% in genus *Leptolyngbya sensu stricto*, 4.64–5.16% in Clade A, and 6.92–9.07% in Clade G. These results indicated that the proportion of SSRs participating CSSR was inconsistent among strains, though similar RA and RD of SSR and CSSR were shared by strains. The significance of CSSR representation, the Z scores, indicated that the nCSSR_obs_ was less than nCSSR_exp_ in 17 genomes, while the opposite results in the remaining 29 genomes. The greatest statistical significance was represented by the genome of *Leptolyngbya* sp. ULC073bin1 (S39).

### 3.3. Distribution and Diversity of SSRs

**Figure 2:**
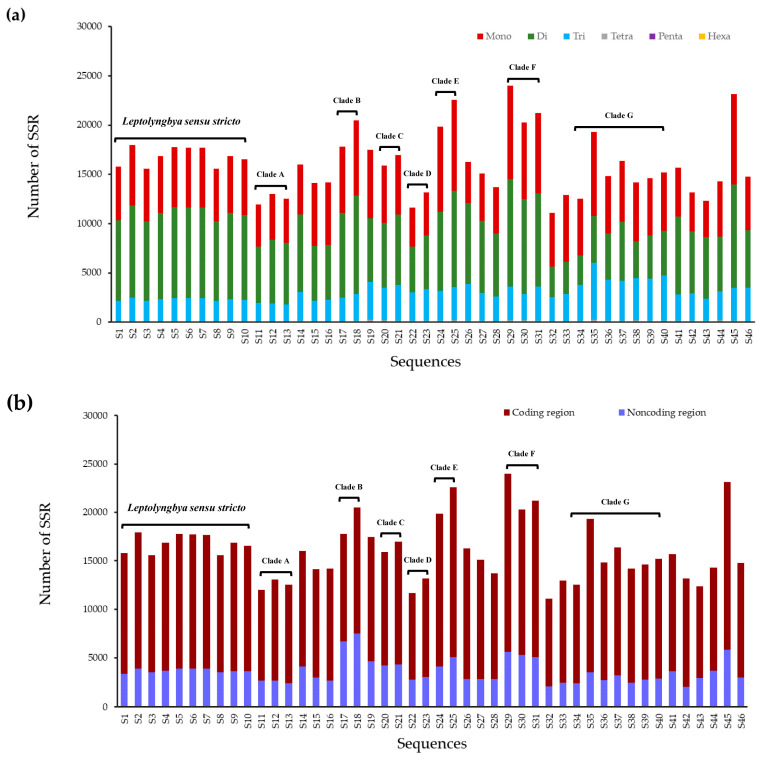
The SSR distribution patterns in the 46 genomes of *Leptolyngbya*-like strains: (**a**) distribution of SSR repeat type; (**b**) SSR distribution in coding and non-coding regions.

As shown in Figure 2a, mononucleotide, dinucleotide, and trinucleotide SSRs accounted for the vast majority of SSRs in each genome, from 98.58% to 99.50%. However, the most abundant category was different among genomes. Dinucleotide SSRs were the most abundant in 31 genomes, accounting for 39.41 to 52.11% of all SSRs, followed by mononucleotide and trinucleotide SSRs, while the other 15 genomes followed the pattern: mono- > di- > trinucleotide SSRs. The proportion of tetranucleotide SSRs was more than that of pentanucleotide and hexanucleotide SSRs in each genome. The same distribution pattern of SSR type exited within genus *Leptolyngbya sensu stricto* and Clade A, B, C, D and F (di- > mono- > trinucleotide SSRs), and within clade G (mono- > di- > trinucleotide SSRs), whereas both patterns within Clade E. Overwhelmingly, SSRs were found to be distributed in coding regions of all 46 genomes analyzed (Figure 2b), accounting for 62.4–84.45% of SSRs. And only low percentages of SSRs (15.55–37.60%) were located in non-coding regions.

**Figure 3:**
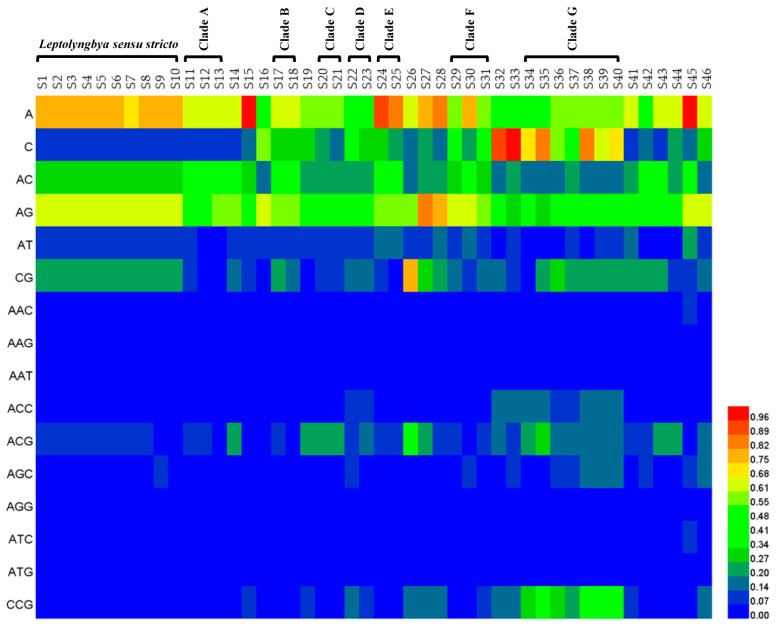
Relative abundance of standard motifs in mononucleotide, dinucleotide and trinucleotide repeat type identified in the 46 genomes of *Leptolyngbya*-like strains.

A heatmap (Figure S1) was constructed to show the relative abundance of 332 standard motifs identified in each genome. There were evident distinctions among genomes regarding relative abundance of motifs in mononucleotide (0.07–1.02), dinucleotide (0.01–0.86) and trinucleotide (0.002–0.999) repeat type (Figure 3). Although genus *Leptolyngbya sensu stricto* showed consistency of the relative abundance of standard motifs, variations of that evidently existed in the clades. The motif (A/C)_n_ was the predominant mononucleotide repeat type in genomes. (AG)_n_, (AC)_n_, and (CG)_n_ were the three most abundant dinucleotide SSRs motifs, among which (AG)_n_ was particularly dominant. Among the trinucleotide repeat type, (ACG)n and (CCG)n were the most abundant motifs. The relative abundances of motifs in tetranucleotide, pentanucleotide, and hexanucleotide repeat types were similar among genomes (Figure S1).

### 3.4. Complexity, Motifs and Distribution of CSSRs

The complexity of CSSRs in the 46 genomes ranged from 2 to 6 (Table S4). A vast majority of complexity was 2, accounting for 93.84% of all the CSSRs (Table S4). And the count of CSSRs decreases with the increase of complexity. Slightly differences of CSSRs complexity were noticed within the genus *Leptolyngbya sensu stricto* or the clades. The complexity of CSSRs for Clade G was up to 6, while that for Clade B was up to 4, and that for the other clades ranged from 2 to 5. These results suggested the diversity of motifs among genomes or groups. Moreover, unique motifs were identified in 39 genomes (Table S5), and the number of unique motifs sharply varied among genomes, from 1 (*L. boryana* PCC 6306) to 84 (*Leptolyngbya* sp. SIO1E4). Unique motifs were identified in only three out of ten strains from genus *Leptolyngbya sensu stricto*.

**Figure 4:**
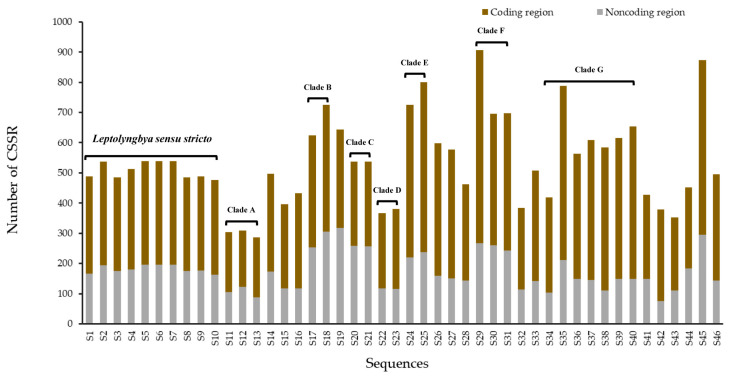
The CSSR distribution in coding and non-coding regions of the 46 genomes of *Leptolyngbya*-like strains.

The distribution of CSSRs, identical to that of SSRs, were also dominantly in coding regions of all 46 genomes analyzed (Figure 4), accounting for 50.6–81.0%. The distribution pattern of SSRs and CSSRs obtained in the present study was in accordance with the prevailing results that SSRs and CSSRs in prokaryotic genomes were located more frequently in coding regions than in non-coding regions [32,33]. Interestingly, it was noticed that the percentage of CSSRs in non-coding regions increased with the increase in complexity.

### 3.5. Correlation Analysis

The Pearson correlation analysis (Table 2) showed that the nSSR was significantly positively correlated with genome size (ρ = 0.81, *p* < 0.01) and negatively correlated with GC content (ρ = −0.30, *p* < 0.05). The nCSSRs correlated positively with genome size (ρ = 0.52, *p* < 0.01), nSSRs (ρ = 0.89, *p* < 0.01) and ncSSR (ρ = 1, *p* < 0.01), but had not significantly correlation with GC content (ρ = 0.11, *p* > 0.05). Therefore, the degree of correlation with nCSSR was ncSSR > nSSR > genome size > GC content.

## 4. Discussion

In this study, bioinformatics tools were employed to provide patterns of distribution, abundance, density, and diversity of SSRs and CSSRs in 46 genomes of *Leptolyngbya*-like strains. The results indicated the dissimilarity patterns of SSRs distribution among these genomes (Table 1, Figure 2), suggesting that SSRs might contribute to the genetic diversity of *Leptolyngbya* genomes and may indicate that they are subject to rapid evolutionary change [34]. The highly consistent patterns of SSRs distribution observed in genus *Leptolyngbya sensu stricto* or the clades implied that the dissimilarity patterns of SSRs distribution were probably ascribed to the genetic discrepancy. The genomes of *Leptolyngbya*-like strains differed in the most abundant repeat type, either mononucleotides or dinucleotides. This was in accordance with the prevalence of mononucleotide or dinucleotide repeats in prokaryotic genomes [35], although sometimes trinucleotide SSRs (e.g., *Cyanobium gracile* PCC 6307) were the most abundant category of SSRs. Mononucleotide repeats were normally characterized as dominant SSRs in eukaryotic genomes, like all human chromosomes [36].

The smaller motifs were predominant in the genomes of *Leptolyngbya*-like strains (Figure S1), and the occurrence decreased with the increase of motif length. This trend was shared in a wide range of organisms [37,38]. The motif (A/C)_n_ were the predominant mononucleotide repeat type in the genomes, which was in agreement with the pattern in other cyanobacteria [35]. Among the dinucleotide SSRs in the genomes, (AG)_n_ was the predominant motif, while the other motifs, like (AT)_n_, were also predominant in cyanobacteria, e.g., *Calothrix*.

The genome sizes of 46 *Leptolyngbya*-like strains ranged from 3.9 Mb to 8.8 Mb (Table 1). The correlation analysis suggested a positively correlation between nSSR/nCSSR and genome size (ρ = 0.81/0.52, *p* < 0.01) (Table 2), although in several cases smaller genomes contained more SSRs or CSSRs (Table 1). The GC content of all the 46 genomes varied from 45.71% to 59.44% (Table 1). Interestingly, GC content had no significant correlation with nCSSR (ρ = 0.18, *p* > 0.05) but negatively significant relation with nSSR (ρ = −0.30, *p* < 0.05). Furthermore, the GC content of SSRs influenced by the GC content of the genome might affect the marker developments due to the difficult amplification of GC-rich SSRs by PCR. In this study, SSRs of 46 genomes of *Leptolyngbya*-like strains appeared to be AT-rich (Figure S1), which might be valuable in the development of SSRs markers.

The complexity analysis of CSSRs in the 46 genomes of *Leptolyngbya*-like strains showed that these CSSRs primarily comprised two SSRs (complexity = 2) (Table S4). As the increase in complexity, the number of CSSRs rapidly decreased. The motifs in CSSRs were quite diverse in each surveyed genome (Table S4). In addition, a large number of unique motifs were identified in 39 genomes (Table S4). These unique motifs were possibly shaped by two reasons. First, the diverse SSR types in each genome generated various motifs (SSR-couple). Second, mutations within SSRs are reported to be frequent [39]. The surveyed *Leptolyngbya*-like strains were from diverse niches (Table 1) and easily possessed diversified mutations during evolutionary processes. This hypothesis was verified by the unique motifs obtained in this study that were differentiated from each other by just one or two single mutations (Table S4).

The SSRs and CSSRs identified in this study were predominantly distributed in coding regions of each genome (Figure 2b and Figure 4). This is probably ascribed to the fact that bacterial genomes are more compact than those of eukaryotes. This result indicated a potential functional role of SSRs and CSSRs in influencing transcription, protein function, gene regulation, and genome organization [9,40]. Particularly, SSRs within genes should be subjected to stronger selective pressure than other genomic regions because of their functional importance [41]. The selection pressure may result in a systematic directional change of the respective repeat number that leads, finally, to desirable activity levels of the adaptationally relevant genes and relaxation of the stress, i.e., adaptation [42]. In this study, a large number of SSRs were identified in the 46 genomes of *Leptolyngbya*-like strains (Table 1). These SSRs may provide an evolutionary advantage of fast adaptation to environmental changes in *Leptolyngbya* and may play an important role in the cosmopolitan distribution of *Leptolyngbya*-like strains to globally diverse niches (Table 1). The different percentages of SSRs distribution in coding regions among groups or phylotypes may suggest a different level of involvement in functions or evolution. However, the possible functions, as well as the mutational mechanism, remain mostly unknown [43]. To date, replication slippage and recombination are currently widely accepted to explain SSR variation. Moreover, overlapping genes extensively existed in prokaryotic genomes, possibly resulting in more influences caused by SSRs or CSSRs. Future studies could increasingly unravel the significant evolutionary role of SSRs in regulating gene expression under diverse environmental stresses.

Variations about genome sizes and distribution patterns of SSRs and CSSRs were evident in the surveyed genomes of *Leptolyngbya*-like strains. This might be attributed to the fact that *Leptolyngbya* has been recognized as polyphyletic [6], and distinct phylotypes existed in the current datasets (Figure 1 and Table S2). Consistent distribution patterns of SSRs and CSSRs were achieved within the genus *Leptolyngbya sensu stricto* or the clades. According to the public microsatellite database (http://big.cdu.edu.cn/psmd/, updated on July 2020, accessed on 20 October 2021), the SSR number of the genomes of *Leptolyngbya*-like strains (11,086 to 24,000) in this study is comparable to that of other cyanobacterial genomes (2283 to 53,041). But evident variations of SSR number were observed at the genus level, such as *Thermosynechococcus* (7490 to 7724) and *Tolypothrix* (32,706 to 37,800). A similar situation was also noticed in CSSR and cSSR% between *Leptolyngbya* genomes and other cyanobacterial genomes. The ANI analysis indicated that several genera might exist among *Leptolyngbya*-like strains (Table S3). This could to some extent explain the observed variations between *Leptolyngbya sensu stricto* and other *Leptolyngbya*-like strains at the genus level. However, the results of phylogenetic and ANI analysis may not be convincing as an ultimately taxonomic assignment given to the polyphyletic traits of *Leptolyngbya*. Recently, polyphasic approaches successfully separated dozens of *Leptolyngbya*-like strains as new genera from *Leptolyngbya sensu stricto* [44,45]. Therefore, reevaluation of these *Leptolyngbya*-like strains is crucial in the future using polyphasic approaches.

Conclusively, a thorough survey was completed to disclose the patterns of distribution, abundance, density, and diversity of SSRs and CSSRs in genomes of *Leptolyngbya sensu stricto* and closely related species. The variations observed are consistent with the consensus that SSRs are generally believed to contribute to genome polymorphism [13]. Besides, the variability of SSR may be considered as one of the drivers of genomic plasticity, thereby allowing targeted mutation and evolution. Further, the identified SSRs may provide an evolutionary advantage of fast adaptation to environmental changes and may play an important role in the cosmopolitan distribution of *Leptolyngbya* strains to globally diverse niches. The current data of SSRs and CSSRs will serve as a prerequisite to facilitate the understanding regarding the genomic distribution, evolution, and functions of SSRs and CSSRs in *Leptolyngbya* genomes.

## Figures and Tables

**Table 1:** Summary of SSRs and CSSRs in 46 genomes of *Leptolyngbya*-like species. Strains were ordered by the appearance on the phylogenetic tree. The strains that were not subjected to phylogenetic analysis were ordered by alphabet.

No.	Species Name	Isolation Source	Size (bp)	GC (%)	SSR			CSSR					
					nSSR ^a^	RA ^b^	RD ^c^	nCSSR ^d^	RA ^b^	RD ^c^	ncSSR ^e^	cSSR% ^f^	Z Score ^g^
S1	*Leptolyngbya boryana* NIES-2135	N/A	6,255,462	47.02	15,780	2.52	16.64	489	0.08	1.05	1006	6.38	0.13
S2	*Leptolyngbya boryana* PCC 6306	USA	7,262,454	47.02	17,953	2.47	16.30	538	0.07	1.01	1110	6.19	0.07
S3	*Leptolyngbya boryana* dg5	N/A	6,176,364	46.99	15,600	2.53	16.67	485	0.08	1.06	1001	6.42	0.06
S4	*Leptolyngbya* sp. FACHB-161	China	6,743,911	46.97	16,858	2.50	16.49	513	0.08	1.03	1061	6.3	0.01
S5	*Leptolyngbya* sp. FACHB-238	Freshwater, China	7,173,154	46.98	17,775	2.48	16.33	539	0.08	1.02	1113	6.27	0.05
S6	*Leptolyngbya* sp. FACHB-239	Terrestrial, China	7,147,343	46.98	17,716	2.48	16.34	539	0.08	1.02	1113	6.29	0.05
S7	*Leptolyngbya* sp. FACHB-402	Freshwater, China	7,138,201	46.98	17,693	2.48	16.34	539	0.08	1.03	1113	6.3	0.05
S8	*Leptolyngbya boryana* IAM M-101	N/A	6,176,363	46.99	15,600	2.53	16.67	485	0.08	1.06	1001	6.42	0.06
S9	*Leptolyngbya* sp. UWPOB_LEPTO1	Activated sludge, Wisconsin, USA	6,800,371	46.86	16,881	2.48	16.36	489	0.07	0.98	1007	5.97	0.11
S10	*Leptolyngbya* sp. FACHB-1624	Freshwater, China	6,648,037	46.89	16,537	2.49	16.41	476	0.07	0.96	972	5.88	0.29
S11	*Leptolyngbya* sp. FACHB-17	Freshwater, China	5,574,121	48.17	11,970	2.15	14.28	303	0.05	0.74	617	5.16	0.28
S12	*Leptolyngbya* sp. NIES-2104	Biofilm, terrestrial, Japan	6,386,309	47.43	13,034	2.04	13.44	309	0.05	0.65	631	4.85	0.23
S13	*Leptolyngbya* sp. NIES-3755	Soil, Japan	6,244,811	46.65	12,513	2.00	13.20	286	0.05	0.61	580	4.64	0.34
S14	*Leptolyngbya* sp. FACHB-321	Terrestrial, China	6,715,002	49.97	15,990	2.38	16.10	497	0.07	1.02	1017	6.37	0.25
S15	*Leptolyngbya* sp. ULC077bin1	Microbial mat, Canada	5,462,880	48.17	14,135	2.59	17.25	397	0.07	0.99	815	5.77	0.16
S16	*Leptolyngbya* sp. ‘hensonii’	Pinnacle phototroph mat, Florida, USA	5,940,029	52.32	14,206	2.39	16.01	433	0.07	1.00	886	6.24	0.23
S17	*Leptolyngbya* sp. FACHB-8	Freshwater, China	6,927,450	50.67	17,792	2.57	16.93	625	0.09	1.21	1295	7.28	−0.03
S18	*Leptolyngbya* sp. FACHB-16	Freshwater, China	8,006,770	50.32	20,514	2.56	16.89	726	0.09	1.22	1509	7.36	−0.12
S19	*Leptolyngbya ohadii* IS1	Biological soil crust, Nitzana, Israel	7,902,459	52.09	17,477	2.21	15.29	644	0.08	1.15	1357	7.77	−0.46
S20	*Leptolyngbya* sp. JSC-1	La Duke Hot Springs, Montana, USA	7,866,824	50.72	15,873	2.09	14.41	537	0.07	1.03	1183	7.46	−1.45
S21	*Leptolyngbya* sp. IPPAS B-1204	Lake water, Lake Tolbo Nuur, Mongolia	8,174,684	50.83	16,980	2.08	14.33	538	0.07	0.96	1165	6.87	−1.05
S22	*Leptolyngbya* sp. DLM2.Bin15	Alkaline salt lake, Cariboo Plateau, Canada	5,006,105	53.93	11,640	2.33	16.29	366	0.07	1.08	759	6.53	−0.04
S23	*Leptolyngbya* sp. CCY15150	North Sea beach, Schiermonikoog, Netherlands	5,756,177	53.40	13,178	2.29	15.98	381	0.07	0.96	781	5.93	0.18
S24	*Leptolyngbya* cf. ectocarpi LEGE 11,479	Diving spot near Leixes Harbour, Portugal	6,774,485	49.35	19,842	2.93	19.64	726	0.11	1.46	1482	7.47	0.37
S25	*Leptolyngbya* sp. Heron Island J	Heron Island, Australia	8,064,167	48.05	22,589	2.80	18.77	800	0.10	1.37	1646	7.29	0.16
S26	*Leptolyngbya* sp. SIO4C1	Stromatolite, Millennium Atoll, Pacific Ocean	5,299,754	54.81	16,272	3.07	21.33	598	0.11	1.63	1240	7.63	−0.05
S27	*Leptolyngbya* sp. BC1307	Lake Hoare margin, microbial mat, Antarctica	4,916,582	52.93	15,073	3.07	20.91	578	0.12	1.64	1180	7.83	0.33
S28	*Leptolyngbya foveolarum* ULC129bin1	Microbial mat, Antarctica	4,750,982	51.01	13,708	2.89	19.52	463	0.10	1.37	955	6.97	0.07
S29	*Leptolyngbya* sp. SIO1E4	Samoa, marine benthic turfs, American	8,792,215	51.44	24,000	2.73	18.19	907	0.10	1.43	1865	7.78	0.19
S30	*Leptolyngbya* sp. SIO1D8	Portobelo marine benthic turfs, Panama	7,757,311	47.92	20,287	2.62	17.41	695	0.09	1.23	1428	7.04	0.19
S31	*Leptolyngbya* sp. SIOISBB	Marine benthic turfs, Indonesia	8,337,037	51.86	21,217	2.55	17.06	698	0.08	1.17	1441	6.8	0.06
S32	*Leptolyngbya* sp. RL_3_1	Stromatolite, Cape Recife, South Africa	3,943,200	56.13	11,086	2.81	19.44	384	0.10	1.40	796	7.19	−0.03
S33	*Leptolyngbya* sp. LCM1.Bin17	Alkaline salt lake, Cariboo Plateau, Canada	4,618,091	55.39	12,930	2.80	19.44	507	0.11	1.60	1050	8.13	−0.02
S34	*Leptolyngbya* sp. BL0902	Imperial Valley, California, USA	4,710,209	57.65	12,530	2.66	19.04	418	0.09	1.31	866	6.92	−0.02
S35	*Leptolyngbya* sp. KIOST-1	Culture pond, Ansan, South Korea	6,320,122	59.44	19,308	3.06	22.05	788	0.12	1.88	1642	8.51	−0.19
S36	*Leptolyngbya* sp. ULC186bin1	Microbial mat, Belgium	5,080,999	57.41	14,809	2.92	20.70	564	0.11	1.66	1185	8.01	−0.36
S37	*Leptolyngbya* sp. FACHB-60	Freshwater, China	5,913,379	55.56	16,386	2.77	19.33	609	0.10	1.48	1246	7.61	0.28
S38	*Leptolyngbya* sp. DLM2.Bin27	Alkaline salt lake, Cariboo Plateau, Canada	4,277,754	59.01	14,185	3.32	24.08	585	0.14	2.06	1214	8.56	−0.07
S39	*Leptolyngbya* sp. ULC073bin1	Microbial mat, Antarctica	4,608,713	57.79	14,600	3.17	22.73	616	0.13	2.03	1312	8.99	−0.72
S40	*Leptolyngbya antarctica* ULC041bin1	Microbial mat, Antarctica	4,711,992	58.22	15,225	3.23	23.31	654	0.14	2.11	1380	9.07	−0.50
S41	*Leptolyngbya* sp. 7M	Thermal spring, Miravalles, Costa Rica	6,990,850	49.92	15,706	2.25	15.09	428	0.06	0.83	875	5.58	0.25
S42	*Leptolyngbya* sp. AVDCRST_MAG94	Avdat LTER, Negev Desert, Israel	5,510,397	52.11	13,152	2.39	16.45	378	0.07	0.98	770	5.86	0.31
S43	*Leptolyngbya* sp. ES-bin-22	Knuths Fjeld, Little Firn glacier, Greenland	5,129,489	51.62	12,342	2.41	16.29	352	0.07	0.96	735	5.96	−0.17
S44	*Leptolyngbya* sp. FACHB-711	Freshwater, China	6,345,842	50.70	14,268	2.25	15.41	452	0.07	1.01	931	6.53	0.10
S45	*Leptolyngbya* sp. SIO3F4	Marine benthic turfs, Boco del Toro, Panama	8,111,628	45.71	23,151	2.85	19.06	873	0.11	1.47	1801	7.78	0.09
S46	*Leptolyngbya* sp. SIO4C5	Stromatolite, Marion Bay Southeast, Australia	5,604,438	53.04	14,754	2.63	18.26	496	0.09	1.28	1027	6.97	−0.01

^a^ nSSR: number of SSRs in each genome; ^b^ RA, relative abundance = number of SSRs or CSSRs per kb; ^c^ RD, relative density is defined as the total length (bp) contributed by each SSR or CSSR per kb of sequence analyzed; ^d^ nCSSR: number of CSSRs in each genome; ^e^ ncSSR, number of cSSR in each genome; ^f^ Percentage of individual SSRs being part of CSSRs (cSSR% = ncSSR/nSSR); ^g^ Z score, statistical significance of CSSR representation. Z > 0, nCSSR_obs_ > nCSSR_exp_; Z < 0, nCSSR_obs_ < nCSSR_exp_. The greater |Z| score is, the greater statistical significance of the CSSR is.

**Table 2:** Correlation analyses between nCSSR and genome size, GC content, nSSR and ncSSR.

		Genome Size	GC Content	nSSR	ncSSR
nSSR	ρ	0.81	−0.30	-	0.88
	Significance	*p* < 0.01	*p* < 0.05	-	*p* < 0.01
nCSSR	ρ	0.52	0.11	0.89	1.00
	Significance	*p* < 0.01	*p* > 0.05	*p* < 0.01	*p* < 0.01

## Data Availability

The data presented in this study are openly available in the National Center for Biotechnology Information (https://www.ncbi.nlm.nih.gov/genome/, accessed on 20 October 2021).

## Supplementary Materials

The following are available online at https://www.mdpi.com/article/10.3390/life11111258/s1, Figure S1: Relative abundance of standard motifs identified in *Leptolyngbya* genomes, Table S1: Information of 53 *Leptolyngbya* genomes retrieved from NCBI, Table S2: Distance matrix of concatenated sequences of 15 genes from 40 *Leptolyngbya* genomes, Table S3: Detailed information on CSSRs identified in 46 Leptolyngbya genomes, Table S4: Unique motifs of CSSRs identified in each genome, Table S5: Unique motifs of CSSR identified in each genome.
