# Supplementary figures and images for: Genome-Wide Investigation and Analysis of Microsatellites and Compound Microsatellites in Leptolyngbya-like Species, Cyanobacteria
Source: Life (Basel). 2021 Nov 18;11(11):1258. doi: 10.3390/life11111258 (PMC8619395; doi:10.3390/life11111258)

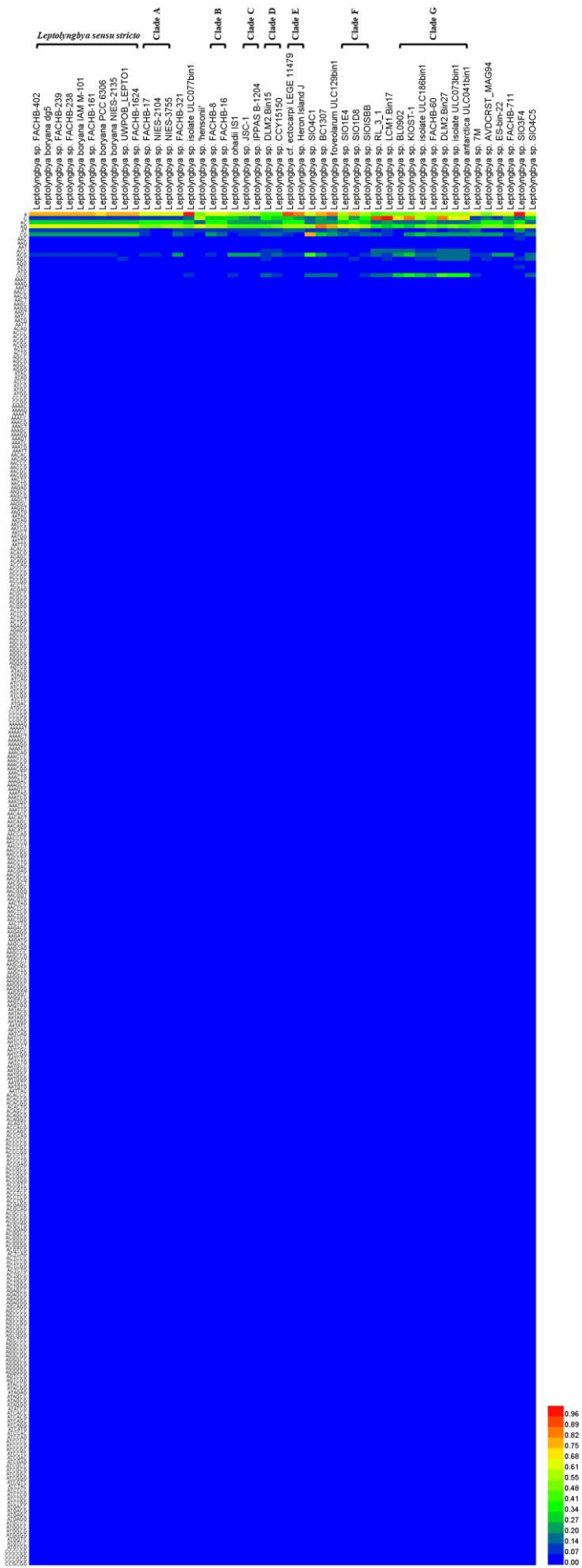

**Figure S1.** Relative abundance of standard motifs identified in *Leptolyngbya* genomes.

Supplement: Supplementary file 1 [file life-11-01258-s001.zip › Figure S1.pdf]
